# Evaluation of a novel real-time polymerase chain reaction assay for identifying H3 equine influenza virus in Kazakhstan

**DOI:** 10.14202/vetworld.2023.1682-1689

**Published:** 2023-08-19

**Authors:** Nurlan Sandybayev, Vitaliy Strochkov, Vyacheslav Beloussov, Shynggys Orkara, Aidyn Kydyrmanov, Yelizaveta Khan, Zhanat Batanova, Markhabat Kassenov

**Affiliations:** 1 Kazakhstan-Japan Innovation Centre, Kazakh National Agrarian Research University, 050010 Almaty, Kazakhstan; 2 TreeGene Molecular Genetics Laboratory, Almaty 050009, Kazakhstan; 3 Research and Production Center for Microbiology and Virology, Almaty 050060, Kazakhstan; 4 Faculty of Veterinary, Kazakh National Agrarian Research University, Almaty 050010, Kazakhstan; 5 Laboratory of Virology, Kazakh Scientific Research Veterinary Institute, Almaty 050016, Kazakhstan

**Keywords:** equine influenza, hemagglutinin, horses, primers, probe, real-time polymerase chain reaction assay, virus

## Abstract

**Background and Aim:**

Equine influenza (EI) is a highly contagious disease that causes fever and upper respiratory tract inflammation. It is caused by influenza virus A, belonging to the *Orthomyxoviridae* family, with subtypes H3N8 and H7N7. This study presents data on the development of a real-time polymerase chain reaction (RT-PCR) assay using TaqMan probes to detect the H3 subtype of EI virus (EIV).

**Materials and Methods:**

The evaluation of the developed RT-PCR assay involved five strains of EIV as positive controls and ten nasopharyngeal swab samples collected from horses. RNA was isolated using the GeneJet Viral DNA and RNA Purification Kit, and primers and probes were designed using the Integrated DNA Technology PrimerQuest Tool. The assay was optimized by investigating the annealing temperature, primer and probes concentrations, sensitivity, and specificity. Sequencing was performed using the Thermo Fisher 3130 Genetic Analyzer, and the evolutionary history was inferred using the Neighbor-Joining method.

**Results:**

The designed primers and probes, targeting the H3 gene, were found to be specific to the EIV. The RT-PCR assay was capable of detecting as low as 50 femtogram (f) or 3 × 10^3^ copies of genomic RNA. No cross-reactions were observed with other respiratory viral and bacterial pathogens, indicating the high specificity of the assay. To evaluate its effectiveness, ten nasopharyngeal swab samples collected from farms in North Kazakhstan regions during disease monitoring were analyzed. The accuracy of the analysis was confirmed by comparing the results with those obtained from a commercial RT-PCR assay for EI identification. The developed RT-PCR assay exhibited high sensitivity and specificity for detecting the EIV.

**Conclusion:**

The results demonstrate that the developed RT-PCR assay is suitable for diagnosing EI. This simple, highly sensitive, and specific assay for detecting H3 EIV can be a reliable tool for diagnosing and surveilling EI. Implementing this RT-PCR assay in veterinary practice will enhance and expedite the timely response to potential outbreaks of EI, thus positively impacting the overall epizootic well-being of EI in Kazakhstan.

## Introduction

Influenza is a widespread infection that affects various species and is characterized by global spread and constant pathogen variability. Equine influenza (EI) is a common acute respiratory infection affecting equids, including horses, donkeys, mules, and zebras, caused by the influenza A virus.

EI is a respiratory infection in horses caused by a virus from the *Orthomyxoviridae* family, specifically the genus A, which spreads rapidly [1, 2]. The influenza virus (IV) is easily transmitted through direct contact and airborne transmission, allowing for rapid spread over long distances. The increasing international relations in sports and horse breeding contribute to the risks of EI dissemination [3].

The high antigenic variability of IV gives it significant potential for interspecies infection, including humans, horses, dogs, cats, birds, marine mammals, bats, and pigs [4–9]. At present, IVs are classified into 16 hemagglutinin (HA) and 9 neuraminidase (NA) subtypes circulating among animals and humans, based on the antigenicity of the two viral surface glycoproteins, HA, and NA [10].

All mammalian IVs can be traced back to a single evolutionary origin associated with the avian reservoir. Among these, specific combinations of HA and NA subtypes have significant importance, such as H3N2 and H1N1 among humans and pigs and H3N8 among horses. In addition, there are increasing reports of EI virus (EIV) H3N8 adapting to dogs [11]. Furthermore, there is evidence of human infection with EIV [12]. The first confirmed cases of human infection resulting from contact with horses were reported in 1957 in Ukraine through serological diagnostics [13].

EI is caused by the H7N7 and H3N8 subtypes of the virus. The EIV was initially isolated and described in 1956 during a significant outbreak in Czechoslovakia [14]. The prototype virus, A/equine/Prague/56, had the antigenic formula H7N7. This virus circulated among the horse population in the Central Asian region until the mid-1970s, with the last outbreak caused by this pathogen being recorded in 1979 [15]. The prototype A/equine/Miami/63 virus entered the susceptible animal population through imported horses from Argentina [16]. The H7N7 virus primarily affects the heart muscle, while the H3N8 virus is more severe and systemic. In 2017, EI was reported in the United Kingdom and the United States. In the US, it is considered endemic in 14 states. In 2018, cases of EI were reported in Ecuador, France, and the UK, where the disease is also considered endemic [17, 18].

Kazakhstan has a longstanding horse breeding tradition, an essential sector of the national economy. As of 2022, the horse population in Kazakhstan reached 3.9 million, indicating positive growth compared to 3.2 million in 2021 [19]. However, expanding the horse population also brings certain risks associated with the spread of infectious diseases among animals.

In the Republic of Kazakhstan, EI is a pressing issue in veterinary medicine. Epizootics of the infection were registered among horses in various regions, including South Kazakhstan, Zhambyl, Almaty, Karaganda, and East Kazakhstan, in 2007 and 2012. Typing and sequencing studies conducted on these strains revealed that they belong to the A/H3N8 subtype of the American genotype, specifically the Florida 2 lineage [20].

To address this issue, implementing quarantine measures for newly imported animals and conducting regular screening using molecular diagnostic methods based on polymerase chain reaction (PCR) are crucial tools for diagnosing infections. Strengthening control over the epizootic situation in border territories is a priority for veterinary services.

The availability of a diagnostic assay based on real-time PCR (RT-PCR) offers significant advantages for effectively controlling EI in Kazakhstan. At present, RT-PCR-based diagnostic methods are considered the most relevant approaches for identifying the causative agent of the disease [21].

This study aimed to develop and evaluate RT-PCR assay to detect H3 EI in Kazakhstan.

## Materials and Methods

### Ethical approval

The study obtained ethical approval from the Local Ethical Committee of LLC SPE “Antigen” on February 08, 2022, with reference number 17.

### Study period and location

The study was conducted from September 2021 to December 2022 at the Kazakhstan-Japan Innovation Centre of Kazakh National Agrarian Research University, Almaty, Kazakhstan.

### Sample collection

Nasopharyngeal swabs were collected from horses in 2022 as part of a scientific project conducted by LLC SPE “Antigen” (unpublished data). The sampling procedure followed the guidance provided by the World Organization for Animal Health [22]. The collected samples were placed in tubes containing medium 199 supplemented with an antibiotic complex (penicillin 2000 U/mL, streptomycin 2 mg/mL, gentamicin 50 μg/mL, and nystatin 50 U/mL) and bovine serum albumin (0.5% w/v). The samples were stored at −80°C until further analysis.

### Viral strains and isolates

RNA from several EIV strains, namely, A/equine/Almaty/24/07 (H3N8), A/equine/Almaty/26/07 (H3N8), A/equine/Almaty/27/07 (H3N8), and A/equine/South Kazakhstan/236/12 (H3N8), was used as a positive control. These strains were obtained from the Research and Production Center for Microbiology and Virology in Almaty, Kazakhstan.

In addition to the EIV strains, RNA from avian influenza (AI) virus isolates of H5 and H9 subtypes and DNA from *Streptococcus* *equi* and *Rhodococcus* *equi* isolates were utilized to evaluate the specificity of the assay.

### RNA isolation

RNA was extracted from nasopharyngeal swabs using the GeneJet Viral DNA and RNA Purification Kit (K0821, ThermoScientific, USA). The concentration and purity of the extracted total RNA were determined using a NanoDrop 2000 spectrophotometer from Thermo Scientific. The isolated RNA was stored at −80°C until further use.

### Complementary DNA (cDNA) synthesis

The high-capacity cDNA Reverse Transcription Kit was employed to synthesize cDNA. The cDNA synthesis reaction mixture consisted of 2 μL of cDNA synthesis buffer (10×), 0.8 μL of 10 mM deoxynucleotide triphosphates (dNTP), 2 μL of Uni12 primer, 1 μL of reverse transcriptase enzyme, 4.2 μL of deionized sterile water, and 10 μL of RNA. The mixture was incubated at 25°C for 10 min, followed by 120 min at 37°C, and finally, 5 min at 85°C. The synthesized cDNA was stored at −20°C.

### Enzyme-linked immunosorbent assay (ELISA) test

An ELISA was performed using the IDEXX influenza A Ab test kit (IDEXX, Montpellier, France) following the instructions provided by the manufacturer.

### Primers and probe selection

Nucleotide sequences of the HA gene of EIV were analyzed to obtain a consensus sequence and identify conserved regions for primer and probe selection. The analysis was conducted using the National Center for Biotechnology Information (NCBI) IV Resource database.

The primers were designed using the Integrated DNA Technology PrimerQuest™ Tool (https://shorturl.at/abkL2). The probe was labeled with fluorescein at the 3’-end, and carboxytetramethylrhodamine dye was attached to the 5’-end (Table-1).

**Table-1:** Primers and probe of the RT-PCR assay for the identification of EIV.

#	Oligonucleotides	Sequence, 5`-3`	Length (bp)
1	EqInf-H3F1(forward)	GAGGTCAATCAGGCAGGATAAG	22
2	EqInf-H3R1 (reverse)	TGGGTGCATCTGATCTCATTAC	22
3	EqInf-H3P1(probe)	FAM-AATGGCAACTTAGTTGCACCGCG-TAMRA	23

RT-PCR=Real-time polymerase chain reaction, EIV=Equine influenza virus

### Optimization of RT-PCR

The RT-PCR assay was optimized by evaluating different parameters and concentration ranges. The annealing temperature was varied from 52°C to 62°C. The concentration of primers for each target gene was tested within the range of 50–800 nM, and the probe concentration was tested between 150 and 250 nM. Optimization involved measuring amplification efficiency and correlation coefficient (R2) and analyzing the amplification curve.

### Determination of RT-PCR sensitivity

The sensitivity of the RT-PCR assay was determined by performing 10-fold serial dilutions of genomic RNA from the H3 subtype of EIV. The dilutions ranged from 5 to 0.00005 ng/μL per reaction, with the genomic RNA of EIV H3 serving as the matrix.

### Determination of RT-PCR specificity

To assess the specificity of the RT-PCR assay, genomic DNA/RNA from closely related viruses and bacterial strains known to cause respiratory diseases in horses were used. A quantity of 5 ng of genomic DNA/RNA from each of these pathogens was included in the specificity analysis.

### Confirmation of RT-PCR suitability

To confirm the suitability of the RT-PCR assay, ten nasopharyngeal swab samples obtained from horses were examined. RNA extraction from these samples was performed using the GeneJet Viral DNA and RNA Purification Kit from ThermoScientific. The viral strain A/equine/Almaty/24/07 (H3N8) was used as a positive control. The results obtained from the RT-PCR analysis were further confirmed using the commercial Oneq PCR-real-time EI H3N8 for the EIV H3N8 detection kit from BioinGentech Biotechnology (Concepción. Chile).

### Sequencing and phylogenetic analysis

The PCR products obtained were purified using the ExoSAP-IT Express PCR Product Cleanup Kit from Thermo Fisher (Santa Clara, USA). The purified PCR products were sequenced using the BigDye Terminator v3.1 Cycle Sequencing kit (Austin, USA), carried out on a 4 capillary 3130 Genetic Analyzer provided by Thermo Fisher.

To analyze the nucleotide sequences, a comparative analysis was conducted using the BLAST program (NCBI, USA), which is available at https://blast.ncbi.nlm.nih.gov/Blast.cgi. The sequences were compared to existing sequences in the database to identify similarities and relationships.

The evolutionary history of the sequences was inferred using the Neighbor-Joining method. The evolutionary distances between sequences were calculated using the Maximum Composite Likelihood method, expressed as the number of base substitutions per site. The analysis included codon positions of 1^st^, 2^nd^, 3^rd^, and non-coding regions. Any ambiguous positions in the sequences were removed (pairwise deletion option) before conducting the analysis.

The phylogenetic analysis was performed using MEGA11 (https://www.megasoftware.net/), a widely used evolutionary analysis tool [23].

## Results

### Sample collection

This study evaluated a new RT-PCR assay for diagnosing EI using samples collected from horses in the North Kazakhstan region. The first appearance of EI in Kazakhstan was recorded in 1992, with a major outbreak observed in several regions, including West Kazakhstan, Aktobe, Atyrau, South Kazakhstan, and Zhambyl [24]. In 2012, local outbreaks of EI occurred in the southern and northern regions of Kazakhstan [25, 26]. Subsequently, in 2020, epizootics of EI induced by influenza A virus H3N8 subtype were registered in the Almaty and Turkistan regions of Kazakhstan, and the corresponding sequences were deposited in GenBank under Accession Numbers OQ815957-OQ815964. In 2022, another outbreak of respiratory disease accompanied by fever, cough, and nasal discharge affected horses on a farm in the North Kazakhstan region [27].

Clinical signs observed in the affected horses included mucous discharge from the nasal openings and eyes, eyelid edema, photophobia, sore throat and larynx, reddened mucous membranes of the eyes and nasal passages, slight swelling covered with clear mucus, submandibular lymph node enlargement, and increased pulse rate and breathing. In total, ten nasopharyngeal swab samples were collected from diseased horses in the North Kazakhstan region for evaluation using the developed RT-PCR assay. The total number of surveyed horse farms and the collected samples are presented in Table-2.

**Table-2:** Sampling of nasopharyngeal swabs from horses in the North Kazakhstan region.

Sample #	Gender	Place of sampling
1	Mare	Zarechniy village
2	Mare	Zarechniy village
3	Mare	Zarechniy village
4	Mare	Zarechniy village
5	Mare	Zarechniy village
6	stallion	Korneevka village
7	Stallion	Korneevka village
8	Stallion	Korneevka village
9	Stallion	Korneevka village
10	Stallion	Korneevka village

### Optimization of the RT-PCR assay

Optimization of the RT-PCR assay involved determining the optimal annealing temperature and the optimal concentrations of primers and probes. The annealing temperature was tested within the range of 52°C–62°C, and it was found that an annealing temperature of 56°C provided the most stable and early amplification curve.

Different concentrations ranging from 50 to 800 nM for primers and 150–250 nM for the probe were tested for the primer and probe concentrations. The optimal concentrations for the TaqMan RT-PCR assay were 400 nM for each primer and 150 nM for the probe (Table-3).

**Table-3:** Optimal conditions of annealing temperature and oligonucleotide concentration for RT-PCR assay.

Primers	Annealing temperature of primers, °C	Concentration of magnesium, mM	Concentration of primers, nM	Concentration of probe EqInf-H3-P1, nM
EqInf-H3F1 EqInf-H3R1	56	2	400/400	150

RT-PCR=Real-time polymerase chain reaction

Based on these optimized conditions, a 20 μL reaction mixture was prepared, consisting of 1× buffer for Taq DNA polymerase, 0.5 μL of 25 mM MgCl_2_, 0.2 mM dNTPs, 1 U of Taq DNA polymerase, 400 nM of each primer, and 150 nM of the probe. The amplification conditions included an initial step at 95°C for 10 s, followed by 45 cycles of 95°C for 10 s, 56°C for 10 s (with fluorescence detection in the green channel), and 72°C for 10 s.

These optimized conditions were used for the subsequent steps of sensitivity determination, specificity testing, and confirmation of the RT-PCR assay using clinical samples.

### Determination of the sensitivity of the developed RT-PCR assay

**Figure-1:**
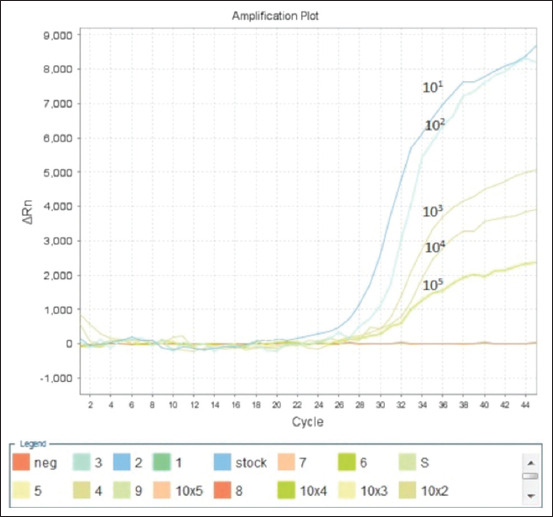
Amplification curves of 10-fold serial dilutions of genomic RNA (5–0.00005 ng/μL per reaction) Equine influenza virus H3.

The analytical sensitivity of the developed RT-PCR assay was determined by testing 10-fold serial dilutions of genomic RNA from EIV H3, ranging from 5 to 0.00005 ng/μL per reaction. Amplification curves for different RNA concentrations are shown in Figure-1. The Ct values obtained for each RNA concentration are presented in Table-4. The assay demonstrated the ability to detect as little as 50 f of genomic RNA from EIV, with Ct values ranging from 20.25 to 28.85.

**Table-4:** Ct values of 10-fold serial dilutions of genomic RNA EIV H.

Dilution of virus RNA	Amount of RNA per reaction, ng	Number of RNA copies	Mean value of Ct ± Standard deviation
stock	5	343,000,000	20.25 ± 0.04
10^-1^	0.5	34,300,000	24.25 ± 0.04
10^-2^	0.05	3,430,000	28.07 ± 0.08
10^-3^	0.005	343,000	28.49 ± 0.10
10^-4^	0.0005	34,300	28.65 ± 0.33
10^-5^	0.00005	3,430	28.85 ± 0.33

### Determination of the specificity of the RT-PCR assay

**Figure-2:**
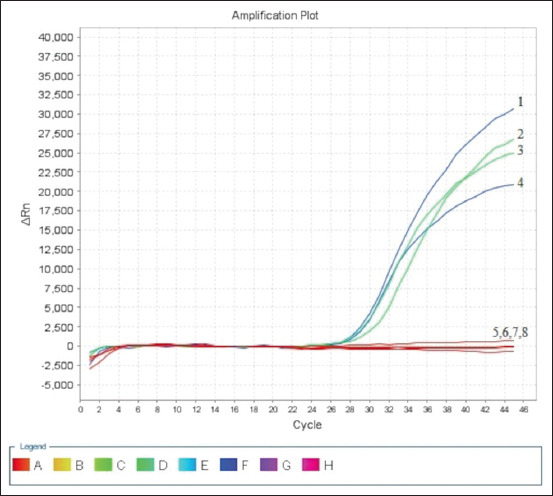
Determination of the specificity of the real-time polymerase chain reaction assay. 1- RNA of equine influenza virus (EIV), A/equine/Almaty/24/07 (H3N8); 2- RNA of EIV, A/equine/Almaty/26/07(H3N8); 3- RNA of EIV, A/equine/Almaty/27/07(H3N8); 4- RNA of EIV, A/equine/South Kazakhstan/236/12(H3N8); 5- RNA of AIV, H5; 6- RNA of AIV, H9; 7- DNA of *Rhodococcus equi*; and 8- DNA of *Streptococcus equi*.

To assess the specificity of the RT-PCR assay, positive controls, including A/equine/Almaty/24/07 (H3N8), A/equine/Almaty/26/07 (H3N8), A/equine/Almaty/27/07 (H3N8), and A/equine/South Kazakhstan/236/12 (H3N8) were used. Negative controls consisted of RNA from AI viruses (H5 and H9) and DNA from bacterial isolates, specifically *S. equi* and *R. equi*. The RT-PCR assay specifically detected the presence of EIV strains. No amplification was observed with the RNA from AI viruses or the DNA from bacterial isolates, indicating the absence of cross-reactivity (Table-5). The assay demonstrated high specificity for EIV strains and no false-positive results. This specificity was confirmed by the absence of amplification signals from other respiratory viruses or bacteria, as shown in Figure-2.

**Table-5:** Evaluation of the specificity of the RT-PCR assay with EIV and other respiratory diseases.

#	RNA and DNA of viruses and bacterium	The diseases they cause	Results of PCR
1	RNA of EIV, strain A/equine/Almaty/24/07 (H3N8)	Equine influenza	Positive
2	RNA of EIV, strain A/equine/Almaty/26/07 (H3N8)	Equine influenza	Positive
3	RNA of EIV, strain A/equine/Almaty/27/07 (H3N8)	Equine influenza	Positive
4	RNA of EIV, strain A/equine/YKO/236/12 (H3N8)	Equine influenza	Positive
5	RNA of AIV, H5	Avian influenza	Negative
6	RNA of AIV, H9	Avian influenza	Negative
7	DNA of *Rhodococcus equi*	Bronchopneumonia in foals	Negative
8	DNA of *Streptococcus equi*	Strangles, an equine respiratory disease	Negative

RT-PCR=Real-time polymerase chain reaction, EIV=Equine influenza virus

### Evaluation of developed RT-PCR assay

The developed RT-PCR assay for EI was evaluated using clinical samples collected from horses in the North Kazakhstan region in 2022. Nasopharyngeal swabs (n = 10) were obtained from horses showing characteristic clinical signs associated with EI. These samples were collected as part of a scientific project by LLC SPE “Antigen” during the monitoring of EI.

In addition to the developed RT-PCR assay, the nasopharyngeal swabs were also tested using the commercial Oneq PCR-real-time EI H3N8 kit for EIV H3N8 detection. The assay was performed according to the manufacturer’s instructions, and the results are summarized in Table-6.

**Table-6:** Evaluation of the developed RT-PCR assay.

# of sample	Place of sampling, village	Results of developed RT-PCR assay	Results of commercial RT-PCR assay	Results of ELISA test
1	Zarechniy	positive	positive	Positive
2	Zarechniy	negative	negative	Positive
3	Zarechniy	positive	positive	Positive
4	Zarechniy	negative	negative	Positive
5	Zarechniy	negative	negative	Positive
6	Korneevka	positive	positive	Positive
7	Korneevka	positive	positive	Positive
8	Korneevka	negative	negative	Positive
9	Korneevka	positive	positive	Negative
10	Korneevka	positive	positive	Negative

RT-PCR=Real-time polymerase chain reaction, ELISA=Enzyme-linked immunosorbent assay

The developed RT-PCR assay detected specific amplicons of EIV in six out of ten nasopharyngeal swabs, resulting in a detection rate of 60% (6/10). Similarly, the commercial Oneq PCR-real-time EI H3N8 kit also identified EIV RNA in six out of ten samples, yielding a detection rate of 60% (6/10).

This comparison indicates that the developed RT-PCR assay performs on par with the commercial kit in terms of detecting EIV in the clinical samples. The results suggest that the developed assay is reliable and can be used effectively to diagnose EI in horses.

In addition, an ELISA test was performed to detect the presence of antibodies to EIV in the collected samples. The results of the ELISA test revealed that antibodies against the virus were detected in eight out of ten samples, indicating a seroprevalence of 80%. However, no antibodies were detected in the remaining two samples (9^th^ and 10^th^).

When comparing the results of the RT-PCR assay, ELISA, and commercial RT-PCR assay, it was found that samples No. 1, 3, 6, and 7 tested positive for both EIV RNA in the RT-PCR assay and antibodies in the ELISA test. These concordant results confirmed the presence of the EIV in these four samples, representing a detection rate of 40% (4/10).

Based on these findings, the developed RT-PCR assay demonstrated high accuracy, specificity, and sensitivity in detecting RNA of the H3 subtype of EIV. It was also suitable for diagnosing EI, yielding similar results to those obtained from the commercial RT-PCR assay. These results validate the effectiveness and reliability of the developed assay for detecting EI in horses.

### Sequencing and phylogenetic analysis

Sequencing was performed on PCR amplification products obtained from four nasopharyngeal swabs (#1, 3, 7, and 8) using the previously mentioned primers (EqInf-H3F1, EqInf-H3R1). The Sanger sequencing method was employed to determine the nucleotide sequences of the amplified regions.

**Figure-3:**
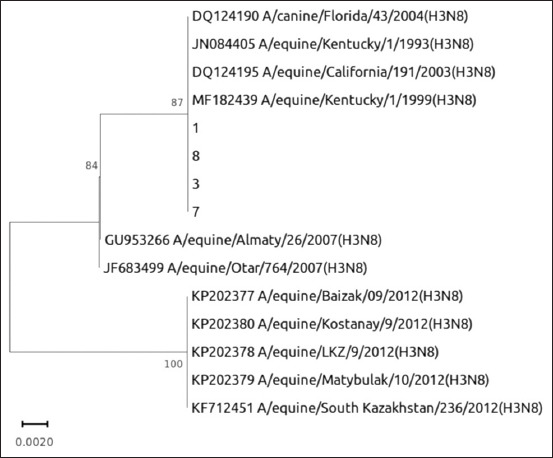
Phylogenetic analysis of the hemagglutinin of the equine influenza virus isolates from the North-Kazakhstan region.

The obtained nucleotide sequences were then subjected to phylogenetic analysis, and the results are presented in Figure-3. The phylogenetic analysis confirmed that the EIVs identified in the nasopharyngeal swabs from horses in the North Kazakhstan region belonged to the H3 subtype. In addition, these viruses exhibited a high degree of genetic similarity to the A/equine/Kentucky/1/1993 (H3N8) strain, which is representative of the Florida 2 lineage.

In comparison to other strains circulating in Kazakhstan, the identified 2022 EIVs were found to be most closely related to the 2007 EIVs, specifically the A/equine/Almaty/26/2007 (H3N8), and A/equine/Otar/764/2007 (H3N8) strains. These findings suggest a genetic continuity and a possible relationship between the viruses circulating in different time periods within the region.

## Discussion

The IV is important in both veterinary and public health due to its high antigenic variability, making it a dangerous pathogen capable of infecting animals and humans. It can cause large epizootics in animals and poses a potential pandemic threat to public health worldwide. Therefore, developing rapid, sensitive, and specific tests to identify the IV is essential for controlling its spread [24].

Direct identification of the pathogen from clinical specimens without prior virus cultivation offers advantages over traditional diagnostic methods. Timely implementation of anti-epizootic measures requires highly sensitive diagnostic assays with high specificity and rapid analysis. Although rapid antigen detection (RAD) tests have been evaluated for diagnosing human influenza, it has been concluded that even RAD tests with the highest sensitivity serve only as an adjunct to molecular tests due to the potential for false-negative results [28].

Real-time polymerase chain reaction assays designed to amplify a region of the EIV matrix gene have been proposed as a more sensitive alternative to virus isolation [29]. However, matrix gene-based RT-PCR assays have limitations in determining the HA subtype of EI. Despite only subtype H3N8 viruses being isolated from horses since 1980, it is still important to determine the HA subtype of EI to exclude infection induced by other subtypes before HA gene sequencing.

At present, expensive imported PCR kits are used in veterinary practice in the Republic of Kazakhstan for diagnosing EIV. The introduction and use of domestically developed assays for diagnosing infectious diseases will significantly reduce costs and expedite the control and response to disease outbreaks. A multiplex RT-PCR test system was developed in Kazakhstan for the simultaneous detection of EI and Equine herpes virus type 1, using primers targeted to the NP gene of local EIV strains and the polymerase gene of equine alphaherpesvirus 1 [30]. This assay was successfully used during a series of EI outbreaks in Kazakhstan but was not applicable for directly confirming the H3 subtype. Therefore, we developed an RT-PCR assay specifically for identifying the H3 subtype of EIV.

During the development of the RT-PCR assay, highly specific primers and a probe were utilized, optimal amplification parameters for the target PCR product were selected, and the assay’s sensitivity and specificity were demonstrated using RNA from EIV strains and clinical samples collected from horses.

To confirm the reliability of the developed assay, positive RNA from four EIV strains was used. All samples tested positive by the RT-PCR assay, and EIV RNA was detected. The specificity of the assay was confirmed by analyzing RNA from closely related viruses and DNA from bacteria known to cause equine respiratory infections, where no specific PCR product was observed.

The sensitivity of the developed RT-PCR assay was determined, and the method was found capable of detecting as low as 50 for 3 × 10^3^ copies of EIV genomic RNA. Similar results have been reported by Aeschbacher *et al*. [31], where the sensitivity ranged from 10^2^ to 10^3^ RNA copies. The high sensitivity of the assay enables the detection of EIV even in small amounts of the virus.

To assess the suitability of the developed RT-PCR assay, ten clinical samples collected during EI monitoring were examined. Equine influenza virus RNA was detected in six out of ten samples. Ghoniem *et al*. [32] evaluated the effectiveness of the developed RT-PCR assay by examining 152 samples obtained from sick horses. In another study by Aeschbacher *et al*. [31], 20 horse samples, 11 pig samples, and two bird samples were used to determine the diagnostic value of the PCR.

To confirm the accuracy of the developed RT-PCR assay, the data from our RT-PCR assay and the commercial Oneq PCR-realtimeTM EI H3N8 for EIV H3N8 detection kit RT-PCR kit (BioinGentech Biotechnology) were compared. Identical results were obtained, as both the commercial kit and the developed RT-PCR assay detected EIV RNA in six out of ten clinical samples.

All ten samples were also analyzed using ELISA (IDEXX Influenza A Ab Test), which showed the presence of antibodies to the influenza A virus in only eight samples. When comparing the RT-PCR and ELISA data, confirmation of influenza A was shown for only four samples. In another four samples, antibodies to influenza A were detected by ELISA, but the virus was not detected by RT-PCR. The condition of the animal can explain this circumstance, perhaps, the horse had previously had the flu, the virus was eliminated, and antibodies may still be present in the blood. Simultaneously, antibodies to the influenza A virus were not found in the two samples, although RT-PCR showed the presence of the virus. We assume that, in this case, the animal may have recently become infected with the flu, and antibodies have not yet been produced.

Phylogenetic analysis of PCR products sequenced in 2022 from nasopharyngeal swabs showed a high degree of homology between the viruses and the A/equine/Kentucky/1/1993 (H3N8) strain. Among the Kazakh strains, the closest matches were the 2007 EIV A/equine/Almaty/26/2007 (H3N8) and A/equine/Otar/764/2007 (H3N8).

## Conclusion

The developed RT-PCR assay for detecting the H3 subtype of EIV represents a valuable addition to the diagnostic tools used for controlling EI in Kazakhstan. The assay demonstrated high sensitivity and specificity, and its utilization offers a feasible and cost-effective alternative to commercial kits. By implementing this assay into routine diagnostic procedures, the risks associated with the spread of EI can be reduced, ultimately safeguarding the horse population. This is particularly significant considering the important role horses play in the national economy and cultural heritage of Kazakhstan.

## Authors’ Contributions

NS, VB, and MK: Conceptualized and designed the study. SO, YK, and ZB: Conducted the laboratory experiments. NS, VS, and AK: Data analysis, writing, reviewing, and editing. All authors have read, reviewed, and approved the final manuscript.
